# Relapsing fever revisited: New insights into neglected borrelial infections

**DOI:** 10.1371/journal.pntd.0014737

**Published:** 2026-09-18

**Authors:** Sally J. Cutler

**Affiliations:** Infection & Immunity Research Group, School of Health, Sport & Bioscience, University of East London, London, United Kingdom; Institute of Continuing Medical Education of Ioannina, GREECE

## Abstract

**Why revisit our understanding of relapsing fever and their causative spirochaetes?** We have seen many existing paradigms of relapsing fever borreliosis challenged over recent years, largely facilitated through technological molecular biological improvements. Concepts that were believed for many years are now being questioned; from the origins of *B. recurrentis*, the epidemic form of relapsing fever, through to the one-tick-one *Borrelia* model. Furthermore, the description of relapsing fever borreliae such as *B. miyamotoi* transmitted by hard tick species and increasing reports of new borrelial species have prompted a re-evaluation of existing knowledge. Within this narrative review, we overview existing knowledge of relapsing fever ecology, epidemiology, diagnostics, disease and prevention, and explore several research gaps worthy of further research.

## Historical perspectives

Descriptions from the time of Hippocrates (430 B.C.) describe ‘yellow fever’, now believed to describe the jaundiced appearance associated with louse-borne relapsing fever (LBRF) [1]. The term relapsing fever was first coined during an outbreak of the louse-borne epidemic form of the disease in Edinburgh 1843–1848 [2]. Despite the recognition of spirochaetes in the blood of patients with clinical relapsing fever in the days of previous eminent researchers such as Otto Obermeier and later Robert Koch [3], these spirochaetal microbes have fascinated and challenged researchers over the years. The genus gained the name ‘Borrelia’ following the mistaken differentiation of ‘*Spirillum gallinarum’* (now known as *Borrelia anserina*) as a peritrichate spiral organism, thus different from treponemes and the non-flexible spirilla by Amédée Borrel [4]. The key for the role of the clothing louse in transmission was then reported by Mackie in 1907 [5].

A recent study used DNA recovered from human remains of those who died from LBRF and were buried at sites across the United Kingdom spanning the last 1,500 years. The researchers investigated the evolutionary divergence of the louse borne *B. recurrentis* from its closest tick-borne relative, *B. duttonii* to determine when and how adaptation from tick-borne to louse-borne transmission occurred [6]. Using molecular clock analyses, they estimated that this transition took place approximately 4000–6000 years ago, coinciding with transition from the Neolithic to Bronze age and increasing adoption of woollen clothing. The adaptation to a new arthropod vector was associated with genomic reduction, a pattern also observed in other pathogens that have switched from tick or flea-borne transmission to lice, including *Bartonella quintana* and *Rickettsia prowazekii* [7,8]. The authors hypothesised that rise of pastoralism in Eastern Europe, together with the adoption of wool for clothing, created conducive ecological conditions for this vector switch [6].

Soft ticks were suggested to have a role in the transmission of an illness (relapsing fever) during the travels of Livingstone published in 1857 [9], but this was not conclusively established until the work of Dutton and Todd, who studied relapsing fever cases in the Congo in 1905 [10]. Similarly, Ross and Milne documented cases in Uganda and Koch studied cases in German East Africa [3,11,12].

Characteristically the louse-borne form of relapsing fever is a disease of poverty, often associated with crowding of people under conditions of poor hygiene and consequently it has been observed during conditions of famine, in prisons, and more recently in refugee camps [13–15]. The global demise of the clothing louse (also known as body lice), *Pediculus humanus*, has resulted in the concomitant reduction of cases of LBRF [16], although recent introductions into Europe with refugees from East Africa, many having passed through Libya [13,17], have demonstrated how cases might still be encountered even in non-endemic regions.

Unlike the epidemic form of relapsing fever described above, tick-borne relapsing fever (TBRF) is considerably more complex. Many causative species show specific associations with particular tick species, with these ticks often defining the geographical distribution of associated TBRF infections seen. Following the established role of soft tick species in transmission of relapsing fever, this was followed by a rapid succession of other tick-borrelial relationships associated with clinical cases. Alongside the elucidation of clinical relapsing fever transmission, a borrelial species, *B. theileri*, was described in 1904 that resembled the human TBRF spirochaetes, but resulted in a relapsing fever presentation in oxen and cattle [18]. Surprisingly, this was associated with hard tick species. Considerably later, we have seen the description of *B. miyamotoi* which can cause a relapsing fever more often in immunocompromised humans, and is phylogenetically clustered amongst the relapsing fever borreliae, but also vectored by hard tick species such as *Ixodes ricinus* and *Ixodes persulcatus* [19].

A recent evaluation of the human clinical burden of relapsing fever estimated that 26,583 cases were reported between January 1874 to December 2022 [20]. This almost certainly underestimates the true burden, owing to underreporting and frequent misdiagnosis of infections.

## Description of pathogen

Relapsing fever borreliae are microaerophilic spirochaetes that can be transmitted via the bite of various Argasid or Ixodid tick species. Remarkably their genome size is relatively small ranging from 1-1.5 Mb and extends beyond the chromosome to a series of both large and small linear plasmids and circular plasmids, that encode several essential genes, thus resembling a ‘segmented genome’ [21]. Most relapsing fever *Borrelia* species infect multiple vertebrate hosts, resulting in complex enzootic transmission cycles and posing a zoonotic risk to humans. A notable exception is *B. duttonii,* which is transmitted by *Ornithodoros moubata* ticks but has become restricted to humans as its sole vertebrate host, as has its human louse-adapted derivative, *B. recurrentis* [6,22]. The type species of the genus is *B. anserina,* which is transmitted by *Argas* ticks, including *A. persicus*. However, the absence of a deposited culture has complicated proteomic, immunological and physiological comparative studies.

Historically the causative borreliae were clustered by geographical location (Old World and New World Borreliae) coupled with tick vectors, however, the characterisation of more recent strains challenges the validity of this geographical grouping. For example, two new *Candidatus* species from Brazil, *Ca*. B. caatinga and *Ca*. B. mimona cluster closely with African and Asian strains [23], conversely, *Ca*. B. kalaharica clusters more with the American species [24]. Characterisation of these pathogens has been severely hampered by difficulties in their cultivation; however, molecular methods have now largely overcome this challenge. Phylogenetic comparison of *Borrelia* spirochaetes has found that they cluster within three main clades with the Lyme borreliosis group (also known as *Borreliella*) that is not discussed further within this review; the relapsing fever group; and metastriate-transmitted borreliae (reptile-associated cluster) [25].

Antigenic variation furnishes these microbes with the ability to evade the vertebrate host immune response and has intrigued researchers over the years. Briefly, this has been most extensively studied in *B. hermsii* where it is facilitated through expression at a telomeric linear plasmid expression site of a particular variable major protein (VMP) within the outer surface membrane (these can be categorised into four sub-groups for the large proteins (VLP) of ~36 kDa and a single group for the small proteins of around 20 kDa (VSP). These spirochaetes possess a library of silent achieved *vmp* alleles that can be rapidly inserted by gene conversion into the expression site by non-reciprocal duplicative recombination resulting in a shift in the expressed VMP within the surface membrane (or serotype) [26], that is often correlated with a clinical relapse of fever and a new wave of spirochaetes present in the blood of their host. It has been suggested that VMP expression in *B. turicatae* correlates with observed tissue tropism of isogenic variants using mouse model [27]. Furthermore, *B. hermsii* has been shown to produce a specific major protein known as variable tick protein (VTP) expressed from its own dedicated promotor whilst within the tick and appears essential for establishment of successful mammalian host infection [28,29].

Studies on antigenic variation for *B. miyamotoi* have revealed that this process involved replacement of up to 16 kb of plasmid sequence from archived linear plasmids. Interestingly, this occurred after just five days post-infection using a mouse model, prior to development of host antibodies, with the lack of antibody derived pressure confirmed by antigenic variation being detected in SCID mice [30].

## Epidemiology

Our understanding of the epidemiology of relapsing fever borreliae has evolved over recent years, assisted by the application of molecular techniques. The belief of ‘one tick one *Borrelia*’ whereby individual tick species were specifically correlated with a single species of *Borrelia* has now been dispelled with some species spanning several tick species. Possibly the greatest myth that we now need to discard is that TBRF is restricted to soft tick species. This is largely a result of our growing recognition of species that cluster within the relapsing fever group such as *B. miyamotoi* and *B. theileri,* but are vectored by various hard tick species. Indeed, hard tick-borne relapsing fever HTBRF species appear to show less stringent spirochaete-tick associations compared with their soft tick-borne counterparts [31].

The human clothing louse, *Pediculus humanus* serves as a vector for several epidemic diseases facilitated by their capacity for dissemination when fleeing from a febrile human host. Louse-borne relapsing fever was once a global epidemic disease, however, it is now restricted to limited regions of East Africa such as Ethiopia, Eritrea, and Somalia [32]. This dramatic reduction reflects the improvements in hygiene and living conditions that have resulted in the demise of the clothing louse [16]. Co-evolution of this pathogen with its louse vector has resulted in its entrapment within an evolutionary bottleneck of human louse transmission. Further reductions in endemic regions are likely to have resulted following largescale use of antibiotics such as those given to treat tropical ocular infections.

In stark contrast to lice, ticks are nidicolous in their behaviour, thus risk of infection occurs when humans have proximity with an enzoonotic transmission cycle such as caves, mud huts, or rustic rural dwellings. Epidemiology of soft tick-borne relapsing fever is dictated by their *Ornithodoros* tick vectors which can be remarkably long-lived, sometimes for 20 years with prolonged periods of starvation yet retain their infectious capacity, thus providing a persistence reservoir [9,33]. Upon acquisition of *Borrelia*, the spirochaete disseminates within the tick sequestering in key sites such as the salivary glands and ovaries that are key locations to ensure onwards transmission [34]. Both transstadial and transovarial tick transmission, bolstered by blood feeding on infected vertebrate hosts maintains the infection, but less conventional routes such as hyperparasitism whereby ticks feed upon engorged ticks, might also contribute to ensuring tick infection [35,36]. The nidicolous nature and short duration of soft tick feeding, typically whilst their host is sleeping, serve to restrict the geographical range of both ticks and their borreliae, unlike hard tick species that can be disseminated further afield whilst feeding on vertebrate host species. Only limited ability of *Ornithodoros* ticks to spread to new areas has been described, largely resulting from combinations of biotic and abiotic factors driven by anthropogenic opportunities [37].

*Borrelia anserina* is the type species for the genus and primarily transmitted by the soft tick *A. persicus* (sometimes *A. miniatus* [38]). This species causes a severe disease in affected poultry associated with high mortality and consequently economic impact but is non-pathogenic in mammalian species. Where tick burdens are particularly high, they will feed on alternative hosts including humans, however, disease consequences attributed to *B. anserina* have not been recorded [39].

America is home to species such as *B. hermsii, B. parkeri* and *B. turicatae* (see S1 Table).

In the United States, during the past decade (2012–2021), a total of 251 cases of soft tick relapsing fever (STRF) have been recorded spanning 11 different states [40]. Cases are typically caused by *B. hermsii* (including the recently reclassified *B. nietonii* – see molecular diagnostics section) or caused by *B. turicatae*. Although *B. parkeri* is also endemic human cases were not recorded within this study [40]. Latin America boasts a rich diversity of soft ticks and their respective relapsing fever borreliae. In Mexico, three species of TBRF have been documented including *B. turicatae; B. dugesi;* and *B. mazzottii* [41].

Moving eastwards to Africa, *B. crocidurae* predominates in west Africa [42], whereas *B. hispanica* predominates in the north (also spilling into southern Europe such as Portugal). *Borrelia duttonii* predominates in the more easterly regions [20] (see S1 Table). Recent descriptions of newer soft tick relapsing fever borreliae include a human case of *Ca*. B. algerica [43], *Ca*. B. fainii recovered from a human and associated with bats in Zambia [44] and *Ca*. B. kalaharica. The latter of these species has resulted in human infection, but only been reported in returning tourists [45,46] leaving its impact upon indigenous populations unexplored.

Moving farther east towards Eurasia we see *B. persica* and countries such as Iran boast incredible STRF diversity with *B. baltazardii, B. latyschewii* and *B. microti*, in addition to *B. persica* [47,48] (see S1 Table). These species demonstrate evolutionary specialisation becoming strictly adapted to their tick vectors raising the suggestion that they are best considered as ecotypes rather than species. There are limited reports of some borreliae in non-classical tick species, but these studies often fail to demonstrate the true competence of that tick species to both harbour and transmit the spirochaete, thus the validity of these reports remains questionable.

Hard tick-borne relapsing fever (HTBRF) includes the recently discovered *B. miyamotoi* which serves as a human pathogen [49] and is transmitted by ticks belonging to the *Ixodes* genus spanning much of Europe, Russia, Japan, and USA [19,50]. Whole genome based studies of 21 isolates of *B. miyamotoi* from diverse geographical locations have confirmed that these cluster as distinct genotypic populations correlated with both their vectors and origins from North America, Asia or Europe [51]. The exception appears to be the Asian genotype that straddles different tick vectors such as *I. persulcatus*, *I. pavlovskyi* and *I. ovatus* ticks in Japan [52], and both *I. ricinus* and *I. persulcatus* in Eurasia [31]. Hard ticks, unlike their soft tick counterparts, attach to their vertebrate host for prolonged periods during feeding and consequently can be dispersed further afield associated with vertebrate host travel. Given the extensive geographical range coupled with a diverse range of vertebrates that can serve as amplification hosts and multiple tick vectors, *B. miyamotoi* displays the greatest breadth compared to all the other relapsing fever species [20].

This spirochaetal group (HTBRF) also includes the often-overlooked veterinary pathogen *B. theileri* known to cause bovine borreliosis, but can also infect other species including camels, deer, horses, sheep, and goats. Like *B. miyamotoi*, this spirochaete appears to have a remarkable geographical distribution ranging from various African nations to the Americas, Bangladesh, Korea, and Thailand, with multiple tick species such as *Rhipicephalus* spp. *Dermacentor* spp. and *Hyalomma* spp. serving as competent vectors [53–56]. *Candidatus* B. lonestari also clusters phylogenetically within HTBRF and has been found associated with ticks and deer in both USA and Japan. Its role as a human pathogen has been an area of controversy and is now believed to lack support. Further strains within the HTBRF group have been reported from *Haemaphysalis* spp. ticks in Japan, but their significance remains to be established [57,58]. In keeping with STBRF, the HTBRF show ability for transovarial transmission that contributes towards their maintenance within tick populations despite often low prevalence of infection [57].

Several species of unknown pathogenicity have been reported from sub-Saharan Africa including *Ca.* B. africana and *Ca.* ivorensis associated with the hard tick *Amblyomma variegatum* collected from Cote d’Ivoire [59]. These borreliae phylogenetically clustered with a strain reported in *A. cohaerens* from Ethiopia [60], but appear distinct from both the Lyme disease and relapsing fever associated strains, thus likely to fall within the metastriate-transmitted (reptile associated) clade of borreliae [25].

## Pathophysiology

For LBRF, transmission does not occur from human to human but requires the intermediary of a clothing louse. These lice only take approximately 1mg of blood, however, during febrile periods this provides a suitable infectious dose for feeding lice [61]. The borreliae are undetectable in the louse for up to 5–6 days but then become evident within the louse [61,62]. Transmission to the human host requires the crushing of the louse into skin abrasions or inoculation of louse faeces [61,62]. Lice are particularly good vectors for epidemic disease transmission given their mass migration from humans spiking a fever facilitating transfer to others in close proximity. Conversely, rather than travelling with their preferred host, ticks tend to be more nidicolous in their lifestyle using ‘sit and wait’ strategy by residing in cracks and crevices until a suitable host becomes available.

The ability of relapsing fever spirochaetes to cause pathology in vertebrate hosts is variable. Species can maintain low levels of spirochaetes in their blood without demonstrable pathological consequences such as chipmunks infected with *B. hermsii* in USA [63]. In highly TBRF endemic regions of Tanzania ambulatory villagers have been found with spirochaetes present in their blood, but without clinical disease [64,65].

The VMPs of LBRF borreliae are instrumental in triggering a specific inflammatory response within the vertebrate host (see above section on description of the pathogen), but might also explain some of the observed clinical heterogeneity seen amongst clinical cases [66]. Furthermore, relapsing fever borreliae have the capacity to bind key regulators of the alternative and lectin complement pathways, specifically factor H and C4b-binding protein, that is likely to facilitate immune evasion thus prolonging survival in the hostile blood stream environment [67]. Similarly to the more classical relapsing fever species, *B. miyamotoi* has been found to bind factor H and C4b-binding protein mediated through complement binding and inhibitory protein A [68].

A phenomenon known as ‘rosetting’ of red blood cells has been described for some members of the group, with this proposed to either be involved in immune evasion, physiology, or serving as a food source for the multiplying spirochaetes. This feature has been suggested to contribute towards clinical severity [69]. This ability is not shared throughout the group, with some such as *B. hispanica, B. duttonii* and *B. persica* showing a marked ability to aggregate red blood cells, whereas others such as *B. recurrentis* or *B. hermsii* cannot [70]. This is believed to be mediated through binding to lactotetraosylceramide, a core structural component of glycoproteins found on erythrocytes and other cell types [69].

The rapid spirochaetal multiplication within the blood makes microscopic visualisation during the febrile periods possible (see diagnostics section). The high blood burden ensures that subsequently feeding vectors acquire infection for onward transmission but also correlates with considerable elevations of TNF-alpha, IL-6, IL-8, and IL-1Beta together with release of inflammatory cytokines and potential disseminated intravascular coagulation. Infection can spread from the blood stream with major organ involvement such as invasion of the brain, myocardium, liver and spleen often associated with haemorrhage, particularly in LBRF [1].

Collectively, our understanding of the virulence factors utilised by the relapsing fever borreliae remains to be elucidated. Research efforts have been hampered by the fastidious nature of these spirochaetes and until recently, the lack of suitable molecular tools such as gene knockout mutants to facilitate our understanding, unlike the Lyme disease associated species [71].

## Clinical manifestations

**Fig 1:**
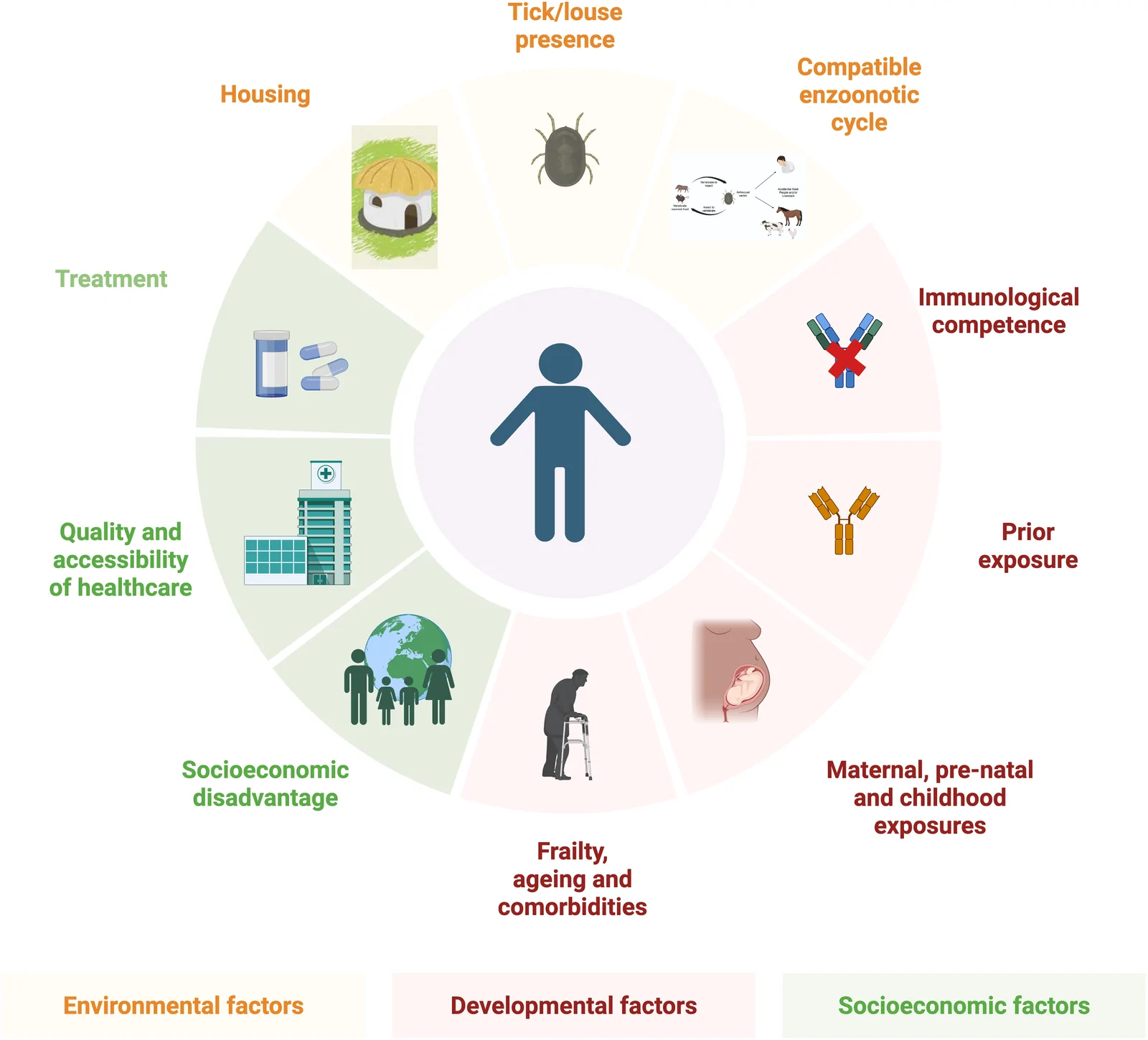
Risk factors for relapsing fever disease severity. The various environmental, developmental, and socioeconomic factors shown influence the probability of infection; disease severity; and clinical outcome. Created in BioRender. Cutler, S. (2026) https://BioRender.com/5a7ahrd.

Clinical disease can vary both in presentation and severity (see Fig 1). The louse-borne form of the disease is deemed to have greatest severity presenting with recurrent fever interspersed by afebrile episodes of several days. In TBRF, cases will present with the abrupt onset of fever following the bite of an infected tick. Febrile episodes are associated with a predominant serotype being present in the blood stream and will last for several days culminating in ‘crisis’ typified by rigors and chills and a period of relative well-being before the onset of another febrile wave associated with a different spirochaetal serotype. Recurrence of febrile episodes varies depending upon the infecting agent with only limited mentions for *B. miyamotoi*, through to 3–5 episodes for LBRF, and up to 13 for some TBRF spirochaetes [26].

The incubation time can vary from just three days to a couple of weeks depending upon the causative species and infective inoculum. The length of the initial febrile attack again varies depending upon the infecting species with TBRF often lasting 3 days whereas this is ~5 days for LBRF [72]. Often fever is coupled with various non-specific symptoms including headache, nausea, vomiting diarrhoea, fatigue, arthralgia and myalgia. After several days of being afebrile (~7 days [range 1–63] for TBRF; and 9 days [range 3–27] for LBRF [72]), a relapse of fever will recur with repeated waves of fever interspersed by afebrile periods. Complications including jaundice, sub-conjunctival haemorrhages, epistaxis and rashes might occur, with greatest severity generally in those with LBRF. Laboratory blood findings often show thrombocytopenia, sometimes with leucocytosis, and raised C-reactive protein [32].

Relapsing fever borreliae, in keeping with other spirochaetal infections, have neurotrophic potential with meningitis approaching 20% of TBRF cases [73]. Remarkably, their presence within the central nervous system tends to be immunologically silent which has led to the hypothesis that this could be an immunologically protected niche within which spirochaetes can persist and undergo antigenic variation (see section above on description of the pathogen) prior to re-seeding the blood stream with the new antigenic variant and the associated relapse of the febrile episodes typical of this disease [74]. Both *B. turicatae* and *B. duttonii* have been linked with neurological involvement [27,74,75]. More recently, meningoencephalitis has been observed following infection with *B. miyamotoi* [76–78].

Relapsing fever is particularly serious during pregnancy (Fig 1) with infection resulting in considerable adverse outcomes including perinatal mortality, low birth weight, and miscarriage. Probably the best studied TBRF during pregnancy is that of *B. duttonii* [79,80]. Animal studies using pregnant mice have confirmed the negative impact on foetal development and demonstrated transplacental transmission [81].

Mortality resulting from relapsing fever most frequently occurs through cardiovascular collapse, but hepatic failure and disseminated intravascular coagulation can also contribute to this outcome. Neurological involvement and intracerebral haemorrhage can also result in death [72].

## Diagnosis

Diagnostics for relapsing fever borreliosis have been challenging, particularly within low- and middle-income countries (LMICs). Demonstration of spirochaetes within the blood has been a key diagnostic approach, however, this is only possible during febrile episodes, and the spirochaetal burden can vary depending upon species. Consequently, alternatives have been sought, but each method has some limitations summarised below.

*Microscopy*: Discovery of this microbe was first achieved by Otto Obermeier whilst tending patients with LBRF during an outbreak in Berlin 1868 [82]. He visualised spirochaetes in blood films taken from febrile patients but also noted that these were not visible during afebrile periods. Visualisation of spirochaetes in the blood requires more than 10^4^ organisms per ml. This has limited the value of microscopy-based diagnostics that both lack sensitivity and fail to discriminate between causative spirochaetes. Improvements have been achieved through direct observation using dark-field microscopy and application of various stains such as Giemsa or Fields stains (or variations upon these). Further enhancement was offered through blood concentration technique that improve sensitivity [83], but this does not overcome the lack of detectable spirochaetes during afebrile periods. Use of dark-field microscopy can improve detection particularly whilst spirochaetes remain motile, but remains insensitive [84].

*Cultivation*: Despite the early microscopic visualisation of relapsing fever borreliae by Otto Obermeier during his studies of the disease in Berlin 1868 [82], cultivation of these fastidious microbes proved challenging. This has hampered research efforts, diagnostics, and epidemiological mapping. Cultivation using axenic medium became a realistic option with the description Kelly’s medium which was used to grow *B. hermsii* in 1971 [85]*.* Successful propagation of adapted strains was possible, but this was less reliable with recovery from the blood of infected mice, prompting refinement undertaken by Stoenner in 1974 [86], accompanied later with the name change of ‘Fortified Kelly medium’. This was further refined to Barbour-Stoenner-Kelly (BSK) which facilitated cultivation of many relapsing fever strains [87] including *B. recurrentis* that had evaded attempts since the days of Robert Koch [88]. This was commercialised as BSK-H [89]. The liquid medium can be adapted to a solidified format where the need for cloning is a priority. This has been used successfully for both *B. hermsii* and *B. turicatae* in a bilayered plate with the upper layer solidified with low melting point agarose [71]. Some species such as *B. miyamotoi* remained largely refractory to cultivation necessitating further media modifications such as BSK-R that is a diluted version of BSK supplemented with Lebovitz’s L15, mouse and foetal bovine serum [90]. Where cultivable isolates have been achieved, matrix-assisted laser desorption time of flight mass spectrometry (MALDI-TOF), has been used successfully to identify borrelial species and can also be used to reveal vector identity using legs removed from the tick vector. Promising research has suggested that these can be combined with different proteomic profiles derived from uninfected and infected ticks, however, no public database is currently available [84].

*Animal Inoculation*: Inoculation of various animals has been pivotal to decipher characteristics of strains in the absence of being able to cultivate them [41], however, conversely the failure of Otto Obermeier to reproduce infection in various animal species hampered the demonstration of infectivity of blood from cases of LBRF [82]. Indeed, findings were often confirmed by inoculation of human volunteers [41]. Inoculation of guinea pigs was pivotal in the differentiation of *B. crocidurae* from *B. duttonii* [72]. In some regions around the globe animal inoculation is still used as a diagnostic method for suspected cases of relapsing fever. Furthermore, this provides a valuable research approach when trying to recover relapsing fever spirochaetes from ticks [41,91]. Feeding ticks upon a vertebrate rodent host allows an amplification of spirochaetal numbers and purification from the complex tick microbiota by recovering from vertebrate blood samples.

*Serology*: Serodiagnosis has classically been used for diagnosis of Lyme borreliosis, and cases subsequently diagnosed as relapsing fever have sometimes achieved their diagnosis through falsely positive Lyme serology. Whole genome analysis of these spirochaetes subsequently revealed key protein differences between the Lyme and relapsing fever borreliae facilitating development of specific serodiagnostic methods for relapsing fever borreliae. Probably the most characterised proteomic differences that can be utilised for specific relapsing fever assays are GlpQ (glycerol phosphodiester phosphodiesterase) first described by Schwan and team in 1996 [92,93] and another differential antigen, BipA (*Borrelia* immunogenic protein A) reported by Lopez and co-workers [94]. GlpQ based assays have been trialled extensively for the diagnosis of *B. miyamotoi* infection, performing better when combined with either a VMP antigen or flagellin [95], but subsequently was noted to suffer with cross-reactivity between *B. miyamotoi* and the C6 antigen of the Lyme borreliae [96]. This spurned further antigen analysis revealed a recombinant BipA capable of differentiating between *B. miyamotoi* and the American soft tick relapsing fever species, thus providing valuable diagnostic potential in areas where these infections co-circulate [97]. Serodiagnostic potential of GlpQ was similarly confirmed for diagnosis of LBRF [98], with its specific performance further enhanced by the addition of the immunogenic N-terminal fragment of CihC protein. This latter protein appears to be absent from other TBRF borreliae, with the notable exception of *B. duttonii* [99].

*Molecular*: As cultivation of relapsing fever borreliae is both low yield and technically challenging, molecular characterisation has been pivotal both for detection and characterisation of these spirochaetes. The high degree of conservation amongst this group has enabled genus specific primers directed against the highly conserved 16S rRNA gene serve as a ‘catch all’ screen. Alternatively, relapsing fever-specific multiplex assays can cluster causative species into clinically relevant groups using a novel conserved gene target (*Bh_0509*) [100]. Unlike many microbes, comparison of 16S RNA has been limited in its discriminatory capacity [101], consequently further species identification requires use of different gene targets. This has typically included flagellin (*flaB*), and glycerophosphodiestase (*glpQ*) with the latter being absent from the Lyme disease associated borreliae. These have been used successfully, but lack the discriminatory value offered by non-coding targets like the 16S-23S rRNA intragenic spacer which has been used to differentiate clonal variants within species [47,102]. This has been further progressed with use of multi-spacer typing [103].

In an attempt to standardise the molecular typing of strains, a multi-locus sequence typing (MLST) approach was developed and has successfully been applied to several representatives of STBRF [104]. The performance of these with some of the more recently described species has not been as robust with several gene targets not showing amplification (such as *nif*S*, pep*X and *uvr*A) [57].

Application of multiple typing methods in combination with whole genomic sequencing has recently demonstrated that the two genomic groups described for *B. hermsii* were more divergent than other ‘sister’ species within the relapsing fever complex such as *B. turicatae* and *B. parkeri*, providing justification for the division of *B. hermsii* into two with genomic group II becoming *B. nietonii* [105]. Conversely, some argue that case for re-classification with many of the current species being regarded as ecotypes within a revised taxonomic classification [33].

## Treatment

Relapsing fever borreliae still remain remarkably susceptible to antibiotics. Treatment of LBRF is usually with tetracycline (or doxycycline), or with penicillin both of which appear similarly effective [106]. It has been proposed that a single dose is sufficient [1], however, longer therapeutic regimes are usually given for both LBRF/TBRF of 7–10 days [73]. Alternative beta-lactams or doxycycline have been used to treat TBRF cases [73], but randomised clinical trials to guide therapeutic decisions are lacking. Some suggest treatment of LBRF should be started with penicillin and later switched to tetracycline as the latter liberates spirochaetal antigens more rapidly, and consequently increases the risk of Jarisch-Herxheimer reaction (JHR) [1,106]. Treatment can be complicated by a JHR with this being particularly severe or even fatal in those with LBRF [1]. A systematic review of 1,189 cases estimated JHR developed amongst 230 (19.3%) of these upon antibiotic treatment [73]. This is marked by a sudden worsening of the patient with a temperature spike, chills, and hypotension. The JHR has been likened to endotoxin reactions accompanied by a proinflammatory cytokine response [73]. Antibodies directed against TNF-alpha can mitigate the JHR among patients with LBRF [107].

Antipyretic supportive management is important particularly during the crisis that signals the end of the febrile phase usually on the fifth day of fever for LBRF, or during subsequent JHR if this occurs. Intravenous isotonic saline and oxygen might be necessary to effectively manage severe cases [1].

In the absence of treatment guidelines for relapsing fever neuroborreliosis, doxycycline and ceftriaxone have both been used successfully for the treatment of *B. crocidurae*-associated neuroborreliosis in returning travellers with treatment ranging from 10 to 21 days [108,109]. Despite frequent delays, often resulting from lack of diagnostic suspicion, full recovery has been reported in these cases. Meningoencephalitis has been described caused by the hard tick relapsing fever species, *B. miyamotoi*, being more frequent in those with immunosuppression. Good therapeutic response was achieved following doxycycline, tetracycline, cephalosporins (including intravenous ceftriaxone), penicillin, or azithromycin, with some cases receiving multiple agents. Ceftriaxone has been recommended as a treatment preference in these cases [110].

## Prevention

Historically, prevention of LBRF was mediated through widespread use of insecticidal dichlorodiphenyltrichloroethane (DDT) [32]. This was then superseded by use of pyrethrin and use of alternative acaricides for reduction of ticks in households [111], but now widespread use of insecticides is more difficult to justify given their biodiversity impacts.

For the louse-borne form of disease, improved hygiene has correlated with massive reductions of clothing louse infestations [112]. As the infectious agent has co-evolved with its louse vector, the demise of clothing lice has curtailed this once epidemic disease. Pockets of infection remain largely centred in Ethiopia and surrounding countries where areas of extreme poverty prevail [113]. The louse is relatively short-lived and cannot survive for extended periods away from its human host, though infested clothing was described associated with an Italian second-hand clothing market suggesting louse survival of some days [114]. A simple and practical solution to reduce clothing louse infestation would be withholding clothes for a period of 10 days, but this depends upon the individual having replacement clothes [115]. Combination of improved living conditions and mass use of antimicrobials in much of sub-Saharan Africa has further added to the reduction in burden of disease caused by *B. recurrentis* as this remains susceptible to both penicillin and tetracyclines [16].

Intervention, reduction and prevention of the tick-borne forms of relapsing fever are more challenging. This results from several factors. Firstly, soft ticks can be particularly long-lived with *O. savignyi* ticks reported to live for 15–20 years with feeding every 5–6 years. During which time they provide a reservoir for infection in subsequent years when transmission opportunities arise. Transovarial transmission or even feeding upon each other (hyperparasitism) under high density conditions [35,36] helps to maintain the borreliae between generations. Secondly, these ticks reside in cracks and crevices of dwellings whether these be mud huts, or infrequently used ‘rustic’ homes, or animal burrows, consequently making it difficult to eradicate infestations. Thorough cleaning of infrequently used holiday accommodation prior to overnight occupation can again mitigate risks for infection [116]. Simple measures such as cementing over cracks and crevices within floors and walls of dwellings have shown benefits in highly endemic regions in Senegal [117]. Others have used insecticide to treat homes in high transmission risk settings with some success [111], but this comes with associated financial and biodiversity costs. Thirdly, these ticks are multi-host feeders and often with diverse host preferences ensuring persistence within their ecological niche through sylvatic cycles. Consequently, prevention is more reliant upon minimising tick exposure. Excluding use of caves during military training exercises has helped reduce occupational risks amongst soldiers in Israel [118], however, bunkers still present infection risks [119].

Precautionary measures for hard tick relapsing fevers are similarly challenging as these ticks reside within the environment with which people have variable contact. General tick bite precautions as advocated for the control and reduction of Lyme borreliosis also reduce risks for exposure to *B. miyamotoi* transmitted by hard tick species [120].

## Current controversies

### When and where did the TBRF, *B. duttonii* adapt to louse transmission and become degraded to *B. recurrentis*?

Recent data have suggested that evolutionary transition from tick transmission to louse might have happened some 6000–4000 years ago and correlated this with the Neolithic to Bronze age adoption of woollen clothing [6].

This raises several important questions, firstly as clothing lice were hypothesised to have evolved from head lice some 170,000–40,000 years ago in Africa [121], although it is thought that this may have occurred on multiple occasions associated with different genotypic louse variants [122]; thus why was the opportunity for louse transmission by *Borrelia* not seized sooner? Secondly, *O. moubata* ticks have not been reported in the Eastern European areas where this switch is believed to have occurred, so how could this vectorial change have occurred in a non-endemic region for *B. duttonii*, the closest relative of *B. recurrentis* [22]? Thirdly, this suggests that an alternative *Borrelia* species and tick vector might have been the ancestral origin for *B. recurrentis*, or that Eastern Europe was not the location for this event, with this happening in a region where *O. moubata* ticks and their *B. duttonii* reside. Interestingly, a *Borrelia* has been reported from Iran that phylogenetically clusters with the *B. recurrentis/B. duttonii* isolates from East Africa [47]. Whether this is a missing link still needs exploration.

### How certain are we regarding current endemic areas for LBRF?

Diagnostic workup on patients with fever in Africa is often not comprehensive because of resource constraints. The recent finding of LBRF amongst refugees trafficking through Libya has raised questions regarding its origin given the timescales of when disease became clinically apparent [123]. Furthermore, the detection of *B. recurrentis* from headlice in a non-endemic region again raises a question over our understanding of the current range of this microbe [124]. Finally, the finding of serological evidence for LBRF amongst febrile individuals residing in northern Kenya is surprising [125]. This study was retrospective, preventing molecular confirmation of the findings and such an assay cannot discriminate between *B. recurentis* and *B. duttonii*, however warrants further investigative studies.

### Could the distribution of the soft tick *O. savignyi* and its *Candidatus* Borrelia kalaharica explain the predicted discrepancy of observed clinical TBRF cases and known endemic regions for the disease?

Recent systematic reviews have helped map the clinical burden of TBRF cases attributed to soft ticks [73]. Here, there appears to be a remarkable gap that resembles the known geographical distribution for the predatory soft tick, *O. savignyi* [126]. This tick has been found to carry a borrelial species *Ca.* B. kalaharica of uncertain pathogenicity that in the absence of current cultivable isolates, remains a Candidatus species. Clinical cases have been recorded in tourists returning from vacations in Africa, however, the potential for infectious burden in clinical or veterinary settings remains to be determined. This is concerning given the far-reaching distribution of *O. savignyi* ticks and their potential for serving as a vector for other pathogens such as Alhumra haemorrhagic fever virus and ticks’ ability for production of salivary toxin [126,127]. This tick was investigated for its potential role as a vector for *B. recurrentis* but was found to be refractory to infection with this species [128].

### Are our beliefs that antigenic variation is driven by development of vertebrate host antibodies outdated?

The dogma that antigenic variation is driven by the development of early antibodies by the vertebrate host specific to the expressed serotype of the dominant infecting antigenic variant has recently been challenged. The report of spontaneous antigenic variation of *B. miyamotoi* within infected SCID mice, provides a challenge to our belief that antigenic variation is driven by antibody-mediated selective pressure [30]. Whether this event is indeed spontaneous or not and if this occurs throughout the relapsing fever group of spirochaetes as a whole warrants further exploration. Indeed, this reawakens the controversies addressed previously by Stoenner and co-workers in the 1980’s who suggested that spontaneous antigenic variation occurred in *B. hermsii* [129].

## Future prospectives

As reviewed above, there are still many knowledge gaps remaining regarding relapsing fever as a clinical entity, and the intricate relationship of these causative spirochaetes and their diverse host species. Clinical burden is most likely hugely underestimated and is hampered by the lack of robust case definitions. Our diagnostic toolkit is evolving, but no single approach can overcome the full range of challenges relapsing fever borreliae present.

The taxonomy and typing of these spirochaetes are complex, with many species assigned only *Candidatus* status because cultivable type strains are unavailable. Furthermore, the presence of species with proposed names lacking formal validation adds to the taxonomic uncertainty and complicates our understanding of this group. As with many infectious diseases, the greatest burden is probably borne by those without resource to combat infection, particularly in low-income settings. Control and intervention strategies are mostly reliant upon preventing tick or louse contact and consequently reducing risk of exposure, however, again this is often reliant upon suitable resources that are often lacking in areas of poverty. The neglect of this group of infections as more of a ‘medical curiosity’ is reflected by lack of substantial research funding that further limits our ability to adequately diagnose, treat and implement sustainable solutions to reduce the burden of disease.

## Supporting information

S1 Table Relapsing fever borreliae and phylogenetically related species including *Candidatus* species. (DOCX)

## Abbreviations

BSK: Barbour-Stoenner-Kelly
HTBRF: Hard tick-borne relapsing fever
JHR: Jarisch-Herxheimer reaction
LBRF: louse-borne relapsing fever
LMICs: low- and middle-income countries
MLST: multi-locus sequence typing
STRF: soft tick relapsing fever
TBRF: tick-borne relapsing fever
VMP: variable major protein
VTP: variable tick protein.
