# Supplementary material for: Relapsing fever revisited: New insights into neglected borrelial infections
Source: PLoS Negl Trop Dis. 2026 Sep 18;20(9):e0014737. doi: 10.1371/journal.pntd.0014737 (PMC13588337; doi:10.1371/journal.pntd.0014737)
Supplement: Supplementary file 1 [file pntd.0014737.s001.docx]

Supplementary Table S1: Relapsing fever borreliae and phylogenetically related species including *Candidatus* species.

|  | *Vector* | Location | Status* | Reference |
| --- | --- | --- | --- | --- |
| *B. anserina* | *Argas ticks* | Global | Valid | [1] |
| *Ca. B. africana* | *A. variegatum* | Cote d'Ivoire | Not valid | [2] |
| *Ca. B. algerica* | Unknown | Algeria | Not valid | [3] |
| *Ca. B. amblyommatis* | *A. javanense* | China | Not valid metastriate group | [4] |
| *B. baltazardii* | *O. tholozani* | Iran | Valid | [5] |
| *B. brasiliensis* | *O. brasiliensis* | Brazil | Valid | [6] |
| *Ca. B. caatinga* | *O. tabajara* | Brazil | Not valid | [7] |
| *B. caucasica* | *O. verrucosus* | Azerbadjan; Georgia; Armenia | Valid | [8] |
| *B. coriaceae* | *O. coriaceus* | USA | Valid | [9] |
| *B. crocidurae* | *O. sonrai* | West Africa | Valid | [10] |
| *B. dugesii* | *O. dugesi* | Mexico | Valid | [11] |
| *B. duttonii* | *O. moubata* | East Africa | Valid | [12] |
| *Ca. B. fainii* | *O. faini* | Zambia | Not valid | [13] |
| *B. graingeri* | *O. graingeri* | Kenya | Valid | [14] |
| *B. hermsii* | *O. hermsi* | USA | Valid | [15] |
| *B. hsipanica* | *O. erraticus; O. marocanus* | Spain; Portugal; north Africa | Valid | [16] |
| *Ca. B. ivorensis* | *A. variegatum* | Cote d'Ivoire | Not valid | [2] |
| *Ca. B. javanense* | *A. javanense* | China | Not valid metastriate group | [4] |
| *Ca. B. johnsonii* | *C. kelleyi* | USA | Not valid | [17] |
| *Ca. B. kalaharica* | *O. savignyi* | Africa | Not valid | [18] |
| *B. latyschewii* | *O. tartakovskyi* | Asia | Valid | [19] |
| *Ca. B. lonestari* | *A. americanum* | USA | Not valid | [20] |
| *Ca. B. mahuryensis* | *A. longirostre; A.geayi* | South America | Not valid metastriate group | [21] |
| *B. mazzottii* | *O. talaje* | Mexico | Valid | [22] |
| *B. microti* | *O. erraticus* | Iran | Not valid | [23] |
| *Ca. B. mimona* | *O. mimon?* | Brazil | Not valid | [24] |
| *B. miyamotoi* | Various *Ixodid* ticks | Global | Valid | [25] |
| *Ca. B. mvumii* | *O. porcinus* | Tanzania | Not valid | [26] |
| *B. nietonii* | *O. hermsi* | USA | Valid | [27] |
| *Ca. B. octodonta* | *O. octodontus* | Chile | Not valid | [28] |
| *B. parkeri* | *O. parkeri* | USA | Valid | [15] |
| *B. persica* | *O. tholozani* | Asia | Valid | [29] |
| *B. puertoricensis* | *O. puertoricensis* | Panama | Not valid | [30] |
| *Ca. B. queenslandica* | Unknown | Australia | Not valid | [31] |
| *B. recurrentis* | *P. humanus* | East Africa (Worldwide) | Valid | [32] |
| *Ca. B. rubricentralis* | Unknown | Indonesia; Australia | Not valid metastriate group | [33] |
| *Ca. B. tachyglossi* | *I. holocyclus* | Australia | Not valid metastriate group | [34] |
| *Ca. B. texasensis* | *D. variabilis* | USA | Not valid | [35] |
| *B. theileri* | *Rhipicephalus & other ticks* | Global | Valid | [36] |
| *B. tillae* | *O. zumpti* | South Africa | Valid | [37] |
| *B. turicica* | *H. aegyptium* | Turkey | Valid | [38] |
| *B. turicatae* | *O. turicata* | USA; Latin America | Valid | [39] |
| *Ca. B. undatumii* | *A. varanense* | Indonesia, Australia | Not valid  metastriate group | [33] |
| *B*. *venezuelensis* | *O. rudis (*formerly *O. venezuelensis)* | Latin America | Valid | [40] |

*= validity according to LPSN.dsmz.de

References:

1.	Sakharoff M. *Spirochaeta anserina* et la septicémie des oies. Ann Inst Pasteur. 1891;5: 564–566.

2.	Ehounoud CB, Yao KP, Dahmani M, Achi YL, Amanzougaghene N, Kacou N’Douba A, et al. Multiple pathogens including potential new species in tick vectors in Côte d’Ivoire. PLoS Negl Trop Dis. 2016;10: e0004367. doi:10.1371/journal.pntd.0004367

3.	Fotso Fotso A, Angelakis E, Mouffok N, Drancourt M, Raoult D. Blood-Borne *Candidatus* Borrelia algerica in a patient with prolonged fever in Oran, Algeria. Am J Trop Med Hyg. 2015;93: 1070–1073. doi:10.4269/ajtmh.15-0124

4.	Jiang B-G, Wu A-Q, Jiang J-F, Yuan T-T, Xu Q, Lv C-L, et al. Molecular detection of novel *Borrelia* species, *Candidatus* Borrelia javanense, in *Amblyomma javanense* Ticks from Pangolins. Pathog Basel Switz. 2021;10. doi:10.3390/pathogens10060728

5.	Karimi Y, Hovind-Hougen K, Birch-Andersen A, Asmar M. *Borrelia persica* and *B. baltazardi* sp. nov.: experimental pathogenicity for some animals and comparison of the ultrastructure. Ann Microbiol (Paris). 1979;130B: 157–168.

6.	Davis GE. Observations on the biology of the argasid tick, *Ornithodoros brasiliensis* Aragão, 1923; with the recovery of a spirochete, *Borrelia brasiliensis*, n. sp. J Parasitol. 1952;38: 473–476.

7.	Oliveira GMB de, Muñoz-Leal S, Santodomingo A, Weck BC, Faccini-Martínez ÁA, Horta MC, et al. A novel relapsing fever group *Borrelia* isolated from *Ornithodoros* ticks of the Brazilian Caatinga. Microorganisms. 2023;11. doi:10.3390/microorganisms11020370

8.	Maruashvili GM. On tick-borne relapsing fever. Med Parazitol (Mosk). 1945;14: 24–27.

9.	Johnson RC, Burgdorfer W, Lane RS, Barbour AG, Hayes SF, Hyde FW. *Borrelia coriaceae* sp. nov.: Putative agent of epizootic bovine abortion. International Journal of Systematic and Evolutionary Microbiology. Microbiology Society; 1987. pp. 72–74. doi:https://doi.org/10.1099/00207713-37-1-72

10.	Leger A. Spirochete de la musaraigne (*Crocidura stampflii)*. Bull Soc Pathol Exot. 1917;10: 280–281.

11.	Mazzotti L. Sobre una nueva espiroqueta de la fiebre recurrente encontrada en México. Rev Inst Salubr Enferm Trop. 1949;10: 277–281.

12.	Novy FG, Knapp RE. Studies on *Spirillum obermeieri* and related organisms. J Infect Dis. 1906;3: 291–393.

13.	Qiu Y, Nakao R, Hang’ombe BM, Sato K, Kajihara M, Kanchela S, et al. Human borreliosis caused by a New World relapsing fever *Borrelia–* like organism in the Old World. Clin Infect Dis. 2018. doi:10.1093/cid/ciy850

14.	Heisch RB, Harvey AEC. The biology of *Ornithodoros graingeri* Heisch & Guggisberg, 1952. Parasitology. 1953;43: 131–132. doi:10.1017/S0031182000018382

15.	Davis GE. Species unity or plurality of the relapsing fever spiroehetes. A symposium on Relapsing Fever in the Americas. Washington DC: American Association for the Advancement of Science; 1942. pp. 41–47.

16.	de Buen E, de la Camara P. Notas sobre 59 casos de fiebre recurrente española. Bol Téc Dir Gen Sanid. 1931;6: 193–207.

17.	Schwan TG, Raffel SJ, Schrumpf ME, Gill JS, Piesman J. Characterization of a novel relapsing fever spirochete in the midgut, coxal fluid, and salivary glands of the bat tick *Carios kelleyi.* Vector Borne Zoonotic Dis Larchmt N. 2009;9: 643–647. doi:10.1089/vbz.2008.0177

18.	Fingerle V, Pritsch M, Wächtler M, Margos G, Ruske S, Jung J, et al. “*Candidatus* Borrelia kalaharica” detected from a febrile traveller returning to Germany from vacation in Southern Africa. PLoS Negl Trop Dis. 2016;10: e0004559. doi:10.1371/journal.pntd.0004559

19.	Sofiev M. *Spirochaeta latyshewi* n. sp. of the recurrens group. Med Parasitol Parasit Dis Mosc. 1941;10: 267–271.

20.	Barbour AG, Maupin GO, Teltow GJ, Carter CJ, Piesman J. Identification of an uncultivable Borrelia species in the hard tick Amblyomma americanum: possible agent of a Lyme disease-like illness. J Infect Dis. 1996;173: 403–409. doi:10.1093/infdis/173.2.403

21.	Binetruy F, Garnier S, Boulanger N, Talagrand-Reboul É, Loire E, Faivre B, et al. A novel *Borrelia* species, intermediate between Lyme disease and relapsing fever groups, in neotropical passerine-associated ticks. Sci Rep. 2020;10: 10596. doi:10.1038/s41598-020-66828-7

22.	Davis GE. A relapsing fever spirochete, *Borrelia mazzottii* (sp. nov.) from *Ornithodoros talaje* from Mexico. Am J Hyg. 1956;63: 13–17. doi:10.1093/oxfordjournals.aje.a119787

23.	Naddaf SR, Ghazinezhad B, Bahramali G, Cutler SJ. Phylogenetic analysis of the spirochete *Borrelia microti*, a potential agent of relapsing fever in Iran. J Clin Microbiol. 2012;50: 2873–2876. doi:10.1128/JCM.00801-12

24.	Weck BC, Santodomingo A, Serpa MCA, de Oliveira GMB, Jorge FR, Muñoz-Leal S, et al. Isolation and molecular characterization of a novel relapsing fever group *Borrelia* from the white-eared opossum *Didelphis albiventris* in Brazil. Curr Res Parasitol Vector-Borne Dis. 2024;6: 100193. doi:10.1016/j.crpvbd.2024.100193

25.	Fukunaga M, Takahashi Y, Tsuruta Y, Matsushita O, Ralph D, McClelland M, et al. Genetic and phenotypic analysis of *Borrelia miyamotoi* sp. nov., isolated from the ixodid tick *Ixodes persulcatus*, the vector for Lyme disease in Japan. Int J Syst Bacteriol. 1995;45: 804–810. doi:10.1099/00207713-45-4-804

26.	Mitani H, Talbert A, Fukunaga M. New World relapsing fever *Borrelia* found in *Ornithodoros porcinus* ticks in Central Tanzania. Microbiol Immunol. 2004;48: 501–505. doi:10.1111/j.1348-0421.2004.tb03545.x

27.	Schwan TG, Raffel SJ, Ricklefs SM, Bruno DP, Martens C. *Borrelia nietonii* sp. nov.: Relapsing fever spirochetes transmitted by the tick *Ornithodoros hermsi* designated previously as *Borrelia hermsii* genomic group II. Vector-Borne Zoonotic Dis. 2024;24: 278–284. doi:10.1089/vbz.2023.0090

28.	Santodomingo A, Thomas R, Thompson M, Robbiano S, Espinoza P, Muñoz-Leal S. Experimental transmission of a novel relapsing fever group *Borrelia* harbored by *Ornithodoros octodontus* (Ixodida: *Argasidae*) in Chile. Exp Appl Acarol. 2024;92: 241–252. doi:10.1007/s10493-023-00881-5

29.	David GE, Hoogstraal  H. Biology of the spirochete *Borrelia persica*, found in the tick *Ornithodorus tholazani* (Argasinae) collected in the governorat of the Western Egyptian Desert; comments on the distribution and ecology of the vector tick. Ann Parasitol Hum Comp. 1956;31: 147–154.

30.	Bermúdez SE, Armstrong BA, Domínguez L, Krishnavajhala A, Kneubehl AR, Gunter SM, et al. Isolation and genetic characterization of a relapsing fever spirochete isolated from *Ornithodoros puertoricensis* collected in central Panama. PLoS Negl Trop Dis. 2021;15: e0009642. doi:10.1371/journal.pntd.0009642

31.	Carley J, Pope J. A new species of *Borrelia* (*B. queenslandica*) from *Rattus villosissimus* in Queensland. Aust J Exp Biol Med Sci. 1962;40: 255–261. doi:10.1038/icb.1962.29

32.	Lebert H, Vogel F. Ziemssen’s Handbuch der Speciellen Pathologie und Therapie. 1874.

33.	Gofton AW, Popa-Baez A, Takano A, Soennichsen K, Michie M, Short M, et al. Characterisation and comparative genomics of three new Varanus-associated *Borrelia* spp. from Indonesia and Australia. Parasit Vectors. 2023;16: 317. doi:10.1186/s13071-023-05937-4

34.	Loh S-M, Gillett A, Ryan U, Irwin P, Oskam C. Molecular characterization of “*Candidatus* Borrelia tachyglossi” (family Spirochaetaceae) in echidna ticks, *Bothriocroton concolor.* Int J Syst Evol Microbiol. 2017;67: 1075–1080. doi:10.1099/ijsem.0.001929

35.	Lin T, Gao L, Seyfang A, Oliver JH. “*Candidatus* Borrelia texasensis”, from the American dog tick *Dermacentor variabilis.* Int J Syst Evol Microbiol. 2005;55: 685–693. doi:10.1099/ijs.0.02864-0

36.	Laveran A. Sur la spirillose des Bovides. Comptes Rendus Académie Sci. 1903;136: 939–941.

37.	Zumpt F, Organ D. Strains of spirochaetes isolated from *Ornithodoros zumpti* Heisch & Guggisberg, and from wild rats in the Cape Province. A preliminary note. South Afr J Lab Clin Med Suid-Afr Tydskr Vir Lab- En Kliniekw. 1961;7: 31–35.

38.	Güner ES, Watanabe M, Hashimoto N, Kadosaka T, Kawamura Y, Ezaki T, et al. *Borrelia turcica* sp. nov., isolated from the hard tick *Hyalomma aegyptium* in Turkey. Int J Syst Evol Microbiol. 2004;54: 1649–1652. doi:10.1099/ijs.0.03050-0

39.	Brumpt E. Étude du Spirochaeta turicatae, n. sp., agent de la fièvre récurrente sporadique des Etats-Unis transmise par Ornithodorus turicata. Comptes Rendus Séances Société Biol. 1933;113: 1369–1372.

40.	Brumpt E. Etude de la fievre recurrente sporadique des etats-unis, transmise dans la nature par *Ornithodoros turicata*. CR Soc Biol. 1933;113: 1366–1369.
